# Deep-learning-assisted diagnosis for knee magnetic resonance imaging: Development and retrospective validation of MRNet

**DOI:** 10.1371/journal.pmed.1002699

**Published:** 2018-11-27

**Authors:** Nicholas Bien, Pranav Rajpurkar, Robyn L. Ball, Jeremy Irvin, Allison Park, Erik Jones, Michael Bereket, Bhavik N. Patel, Kristen W. Yeom, Katie Shpanskaya, Safwan Halabi, Evan Zucker, Gary Fanton, Derek F. Amanatullah, Christopher F. Beaulieu, Geoffrey M. Riley, Russell J. Stewart, Francis G. Blankenberg, David B. Larson, Ricky H. Jones, Curtis P. Langlotz, Andrew Y. Ng, Matthew P. Lungren

**Affiliations:** 1 Department of Computer Science, Stanford University, Stanford, California, United States of America; 2 Quantitative Sciences Unit, Department of Medicine, Stanford University, Stanford, California, United States of America; 3 Department of Radiology, Stanford University, Stanford, California, United States of America; 4 Department of Orthopedic Surgery, Stanford University, Stanford, California, United States of America; Johns Hopkins University, UNITED STATES

## Abstract

**Background:**

Magnetic resonance imaging (MRI) of the knee is the preferred method for diagnosing knee injuries. However, interpretation of knee MRI is time-intensive and subject to diagnostic error and variability. An automated system for interpreting knee MRI could prioritize high-risk patients and assist clinicians in making diagnoses. Deep learning methods, in being able to automatically learn layers of features, are well suited for modeling the complex relationships between medical images and their interpretations. In this study we developed a deep learning model for detecting general abnormalities and specific diagnoses (anterior cruciate ligament [ACL] tears and meniscal tears) on knee MRI exams. We then measured the effect of providing the model’s predictions to clinical experts during interpretation.

**Methods and findings:**

Our dataset consisted of 1,370 knee MRI exams performed at Stanford University Medical Center between January 1, 2001, and December 31, 2012 (mean age 38.0 years; 569 [41.5%] female patients). The majority vote of 3 musculoskeletal radiologists established reference standard labels on an internal validation set of 120 exams. We developed MRNet, a convolutional neural network for classifying MRI series and combined predictions from 3 series per exam using logistic regression. In detecting abnormalities, ACL tears, and meniscal tears, this model achieved area under the receiver operating characteristic curve (AUC) values of 0.937 (95% CI 0.895, 0.980), 0.965 (95% CI 0.938, 0.993), and 0.847 (95% CI 0.780, 0.914), respectively, on the internal validation set. We also obtained a public dataset of 917 exams with sagittal T1-weighted series and labels for ACL injury from Clinical Hospital Centre Rijeka, Croatia. On the external validation set of 183 exams, the MRNet trained on Stanford sagittal T2-weighted series achieved an AUC of 0.824 (95% CI 0.757, 0.892) in the detection of ACL injuries with no additional training, while an MRNet trained on the rest of the external data achieved an AUC of 0.911 (95% CI 0.864, 0.958). We additionally measured the specificity, sensitivity, and accuracy of 9 clinical experts (7 board-certified general radiologists and 2 orthopedic surgeons) on the internal validation set both with and without model assistance. Using a 2-sided Pearson’s chi-squared test with adjustment for multiple comparisons, we found no significant differences between the performance of the model and that of unassisted general radiologists in detecting abnormalities. General radiologists achieved significantly higher sensitivity in detecting ACL tears (*p-*value = 0.002; *q-*value = 0.019) and significantly higher specificity in detecting meniscal tears (*p-*value = 0.003; *q-*value = 0.019). Using a 1-tailed *t* test on the change in performance metrics, we found that providing model predictions significantly increased clinical experts’ specificity in identifying ACL tears (*p-*value < 0.001; *q-*value = 0.006). The primary limitations of our study include lack of surgical ground truth and the small size of the panel of clinical experts.

**Conclusions:**

Our deep learning model can rapidly generate accurate clinical pathology classifications of knee MRI exams from both internal and external datasets. Moreover, our results support the assertion that deep learning models can improve the performance of clinical experts during medical imaging interpretation. Further research is needed to validate the model prospectively and to determine its utility in the clinical setting.

## Abstract 2

Nicholas Bien and colleagues present an automated system for interpreting knee magnetic resonance (MR) images to prioritize high-risk patients and assist clinicians in making diagnoses.

## Author summary

**Why was this study done?:**

- We wanted to see if a deep learning model could succeed in the clinically important task of detecting disorders in knee magnetic resonance imaging (MRI) scans.
- We wanted to determine whether a deep learning model could improve the diagnostic accuracy, specificity, or sensitivity of clinical experts, including general radiologists and orthopedic surgeons.

**What did the researchers do and find?:**

- Our deep learning model predicted 3 outcomes for knee MRI exams (anterior cruciate ligament [ACL] tears, meniscal tears, and general abnormalities) in a matter of seconds and with similar performance to that of general radiologists.
- We experimented with providing model outputs to general radiologists and orthopedic surgeons during interpretation and observed statistically significant improvement in diagnosis of ACL tears with model assistance.
- When externally validated on a dataset from a different institution, the model picked up ACL tears with high discriminative ability.

**What do these findings mean?:**

- Deep learning has the potential to provide rapid preliminary results following MRI exams and improve access to quality MRI diagnoses in the absence of specialist radiologists.
- Providing clinical experts with predictions from a deep learning model could improve the quality and consistency of MRI interpretation.

## Introduction

Magnetic resonance imaging (MRI) of the knee is the standard-of-care imaging modality to evaluate knee disorders, and more musculoskeletal (MSK) MRI examinations are performed on the knee than on any other region of the body [1–3]. MRI has repeatedly demonstrated high accuracy for the diagnosis of meniscal and cruciate ligament pathology [4–7] and is routinely used to identify those who would benefit from surgery [8–10]. Furthermore, the negative predictive value of knee MRI is nearly 100%, so MRI serves as a noninvasive method to rule out surgical disorders such as anterior cruciate ligament (ACL) tears [11]. Due to the quantity and detail of images in each knee MRI exam, accurate interpretation of knee MRI is time-intensive and prone to inter- and intra-reviewer variability, even when performed by board-certified MSK radiologists [12]. An automated system for interpreting knee MRI images has a number of potential applications, such as quickly prioritizing high-risk patients in the radiologist workflow and assisting radiologists in making diagnoses [13]. However, the multidimensional and multi-planar properties of MRI have to date limited the applicability of traditional image analysis methods to knee MRI [13,14].

Deep learning approaches, in being able to automatically learn layers of features, are well suited for modeling the complex relationships between medical images and their interpretations [15,16]. Recently, such approaches have outperformed traditional image analysis methods and enabled significant progress in medical imaging tasks, including skin cancer classification [17], diabetic retinopathy detection [18], and lung nodule detection [19]. Prior applications of deep learning to knee MRI have been limited to cartilage segmentation and cartilage lesion detection [20–22].

In this study, we present MRNet, a fully automated deep learning model for interpreting knee MRI, and compare the model’s performance to that of general radiologists. In addition, we evaluate changes in the diagnostic performance of clinical experts when the automated deep learning model predictions are provided during interpretation. Finally, we evaluate our model’s performance on a publicly available external dataset of knee MRI exams labeled for ACL injury.

## Methods

### Dataset

Reports for knee MRI exams performed at Stanford University Medical Center between January 1, 2001, and December 31, 2012, were manually reviewed in order to curate a dataset of 1,370 knee MRI examinations. The dataset contained 1,104 (80.6%) abnormal exams, with 319 (23.3%) ACL tears and 508 (37.1%) meniscal tears. ACL tears and meniscal tears occurred concurrently in 194 (38.2%) exams. The most common indications for the knee MRI examinations in this study included acute and chronic pain, follow-up or preoperative evaluation, injury/trauma, and other/not provided. Examinations were performed with GE scanners (GE Discovery, GE Healthcare, Waukesha, WI) with standard knee MRI coil and a routine non-contrast knee MRI protocol that included the following sequences: coronal T1 weighted, coronal T2 with fat saturation, sagittal proton density (PD) weighted, sagittal T2 with fat saturation, and axial PD weighted with fat saturation. A total of 775 (56.6%) examinations used a 3.0-T magnetic field; the remaining used a 1.5-T magnetic field. See S1 Table for detailed MRI sequence parameters. For this study, sagittal plane T2-weighted series, coronal plane T1-weighted series, and axial plane PD-weighted series were extracted from each exam for use in the model. The number of images in these series ranged from 17 to 61 (mean 31.48, SD 7.97).

**Fig 1:**
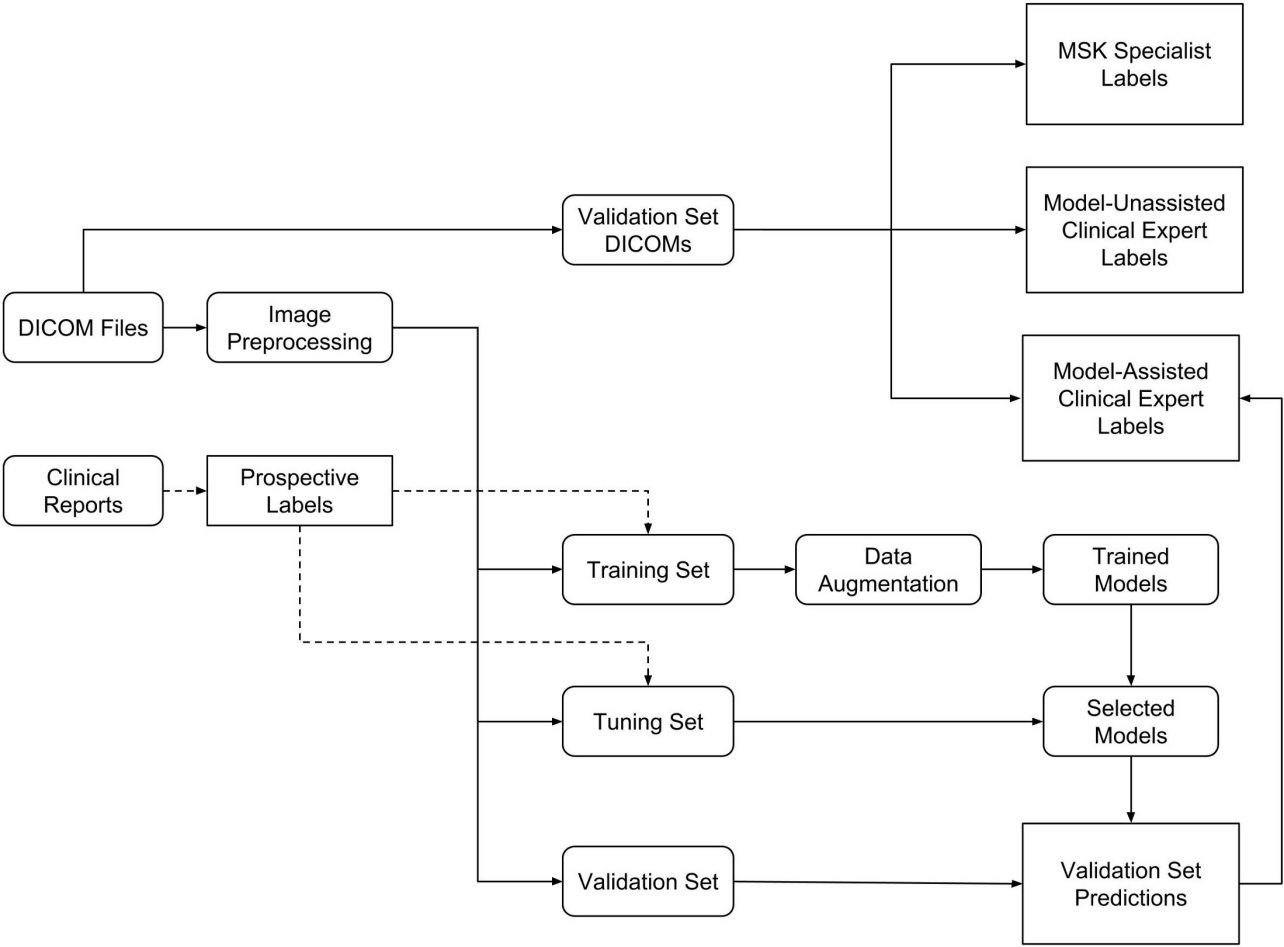
Experimental setup flowchart. We retrospectively collected a dataset of 1,370 knee MRI examinations used to develop the model and to assess the model and clinical experts. Labels were prospectively obtained through manual extraction from clinical reports. Images were extracted from DICOM files, preprocessed, then linked to reports. The dataset was split into a training set (to develop the model), a tuning set (to choose among models), and a validation set (to assess the best model and clinical experts). The validation set DICOMs correspond to the same exams as the validation set, but the images in the validation set were preprocessed before input to the model. These validation exams were independently annotated by musculoskeletal (MSK) radiologists (MSK specialists), model-unassisted clinical experts, and model-assisted clinical experts.

The exams were split into a training set (1,130 exams, 1,088 patients), a tuning set (120 exams, 111 patients), and a validation set (120 exams, 113 patients) (Fig 1). To form the validation and tuning sets, stratified random sampling was used to ensure that at least 50 positive examples of each label (abnormal, ACL tear, and meniscal tear) were present in each set. All exams from each patient were put in the same split. Table 1 contains pathology and patient demographic statistics for each dataset.

**Table 1:** Summary statistics of training, tuning, and validation datasets.

Statistic	Training	Tuning	Validation		
			All	Prospective labels^1^	Reference standard labels^2^
Number of exams	1,130	120	120		
Number of patients	1,088^3^	111	113		
Number of female patients (%)	480 (42.5)	50 (41.7)	39 (32.5)		
Age, mean (SD)	38.3 (16.9)	36.3 (16.9)	37.1 (14.8)		
Number with abnormality (%)	913 (80.8)	95 (79.2)		96 (80.0)	99 (82.5)
Number with ACL tear (%)	208 (18.4)	54 (45.0)		57 (47.5)	58 (48.3)
Number with meniscal tear (%)	397 (35.1)	52 (43.3)		59 (49.2)	65 (54.2)
Number with ACL and meniscal tear (%)	125 (11.1)	31 (25.8)		38 (31.7)	40 (33.3)

The training set was used to optimize model parameters, the tuning set to select the best model, and the validation set to evaluate the model’s performance.

^1^From clinical reports.

^2^From musculoskeletal radiologists.

^3^For 1,114 (98.6%) exams in the training set with patient identifier available.

ACL, anterior cruciate ligament.

### External validation

We obtained a publicly available dataset from Štajduhar et al. [23] consisting of 917 sagittal PD-weighted exams from a Siemens Avanto 1.5-T scanner at Clinical Hospital Centre Rijeka, Croatia. From radiologist reports, the authors had extracted labels for 3 levels of ACL disease: non-injured (690 exams), partially injured (172 exams), and completely ruptured (55 exams). We split the exams in a 60:20:20 ratio into training, tuning, and validation sets using stratified random sampling. We first applied MRNet without retraining on the external data, then subsequently optimized MRNet using the external training and tuning sets. The classification task was to discriminate between non-injured ACLs and injured ACLs (partially injured or completely torn).

### Model

#### Preprocessing

Images were extracted from Digital Imaging and Communications in Medicine (DICOM) files, scaled to 256 × 256 pixels, and converted to Portable Network Graphics (PNG) format using the Python programming language (version 2.7) [24] and the pydicom library (version 0.9.9) [25].

To account for variable pixel intensity scales within the MRI series, a histogram-based intensity standardization algorithm was applied to the images [26]. For each series, a representative intensity distribution was learned from the training set exams. Then, the parameters of this distribution were used to adjust the pixel intensities of exams in all datasets (training, tuning, and validation). Under this transformation, pixels with similar values correspond to similar tissue types. After intensity standardization, pixel values were clipped between 0 and 255, the standard range for PNG images.

#### MRNet

**Fig 2:**
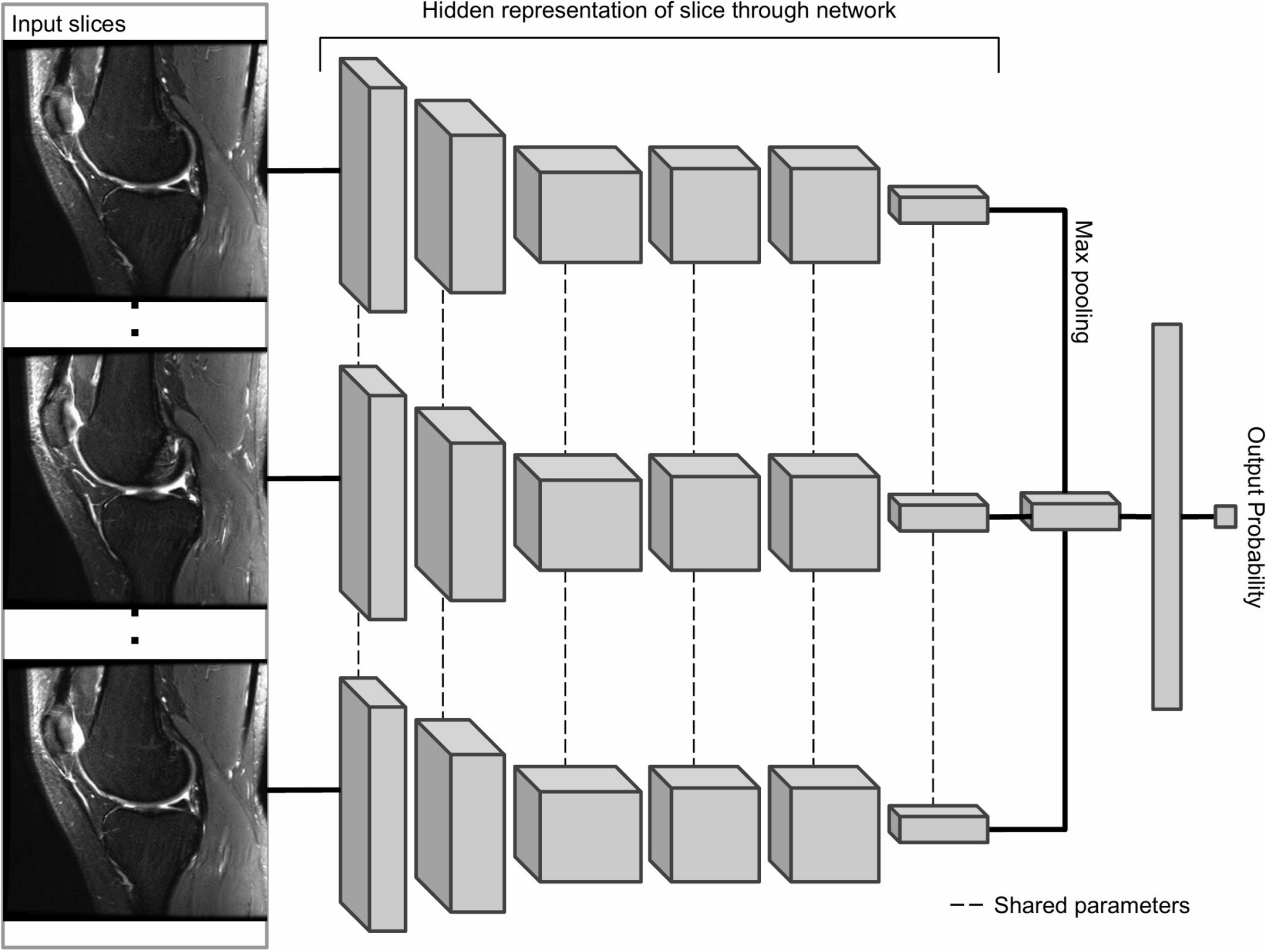
MRNet architecture. The MRNet is a convolutional neural network (CNN) that takes as input a series of MRI images and outputs a classification prediction. AlexNet features from each slice of the MRI series are combined using a max pooling (element-wise maximum) operation. The resulting vector is fed through a fully connected layer to produce a single output probability. We trained a different MRNet for each task (abnormality, anterior cruciate ligament [ACL] tear, meniscal tear) and series type (sagittal, coronal, axial), resulting in 9 different MRNets (for external validation, we use only the sagittal plane ACL tear MRNet). For each model, the output probability represents the probability that the model assigns to the series for the presence of the diagnosis.

The primary building block of our prediction system is MRNet, a convolutional neural network (CNN) mapping a 3-dimensional MRI series to a probability [15] (Fig 2). The input to MRNet has dimensions *s* × 3 × 256 × 256, where *s* is the number of images in the MRI series (3 is the number of color channels). First, each 2-dimensional MRI image slice was passed through a feature extractor based on AlexNet to obtain a *s* × 256 × 7 × 7 tensor containing features for each slice. A global average pooling layer was then applied to reduce these features to *s* × 256. We then applied max pooling across slices to obtain a 256-dimensional vector, which was passed to a fully connected layer and sigmoid activation function to obtain a prediction in the 0 to 1 range. We optimized the model using binary cross-entropy loss. To account for imbalanced class sizes on all tasks, the loss for an example was scaled inversely proportionally to the prevalence of that example’s class in the dataset.

During training, the gradient of the loss was computed on each training example using the backpropagation algorithm, and MRNet’s parameters were adjusted in the direction opposite the gradient [15]. Each training example was rotated randomly between –25 and 25 degrees, shifted randomly between –25 and 25 pixels, and flipped horizontally with 50% probability whenever it appeared in training. Model parameters were saved after every full pass through the training set, and the model with the lowest average loss on the tuning set was chosen for evaluation on the validation set. Fig 2 describes the MRNet architecture in more detail. Training each MRNet for 50 iterations through the training set took 6 hours on average with an NVIDIA GeForce GTX 1070 8GB GPU. MRNet was implemented with Python 3.6.3 [27] and PyTorch 0.3.0 [28].

Training a CNN for image classification from scratch typically requires a dataset larger than 1,130 examples. For this reason, we initialized the weights of the AlexNet portion of the MRNet to values optimized on the ImageNet database [29] of 1.2 million images across 1,000 classes, then fine-tuned these weights to fit our MRI dataset. This allowed the earlier layers of the network, which are more difficult to optimize than later layers, to immediately recognize generic features such as lines and edges. This “transfer learning” approach has similarly been applied to skin cancer [17] and diabetic retinopathy [18] image datasets.

#### MRNet interpretation

**Fig 3:**
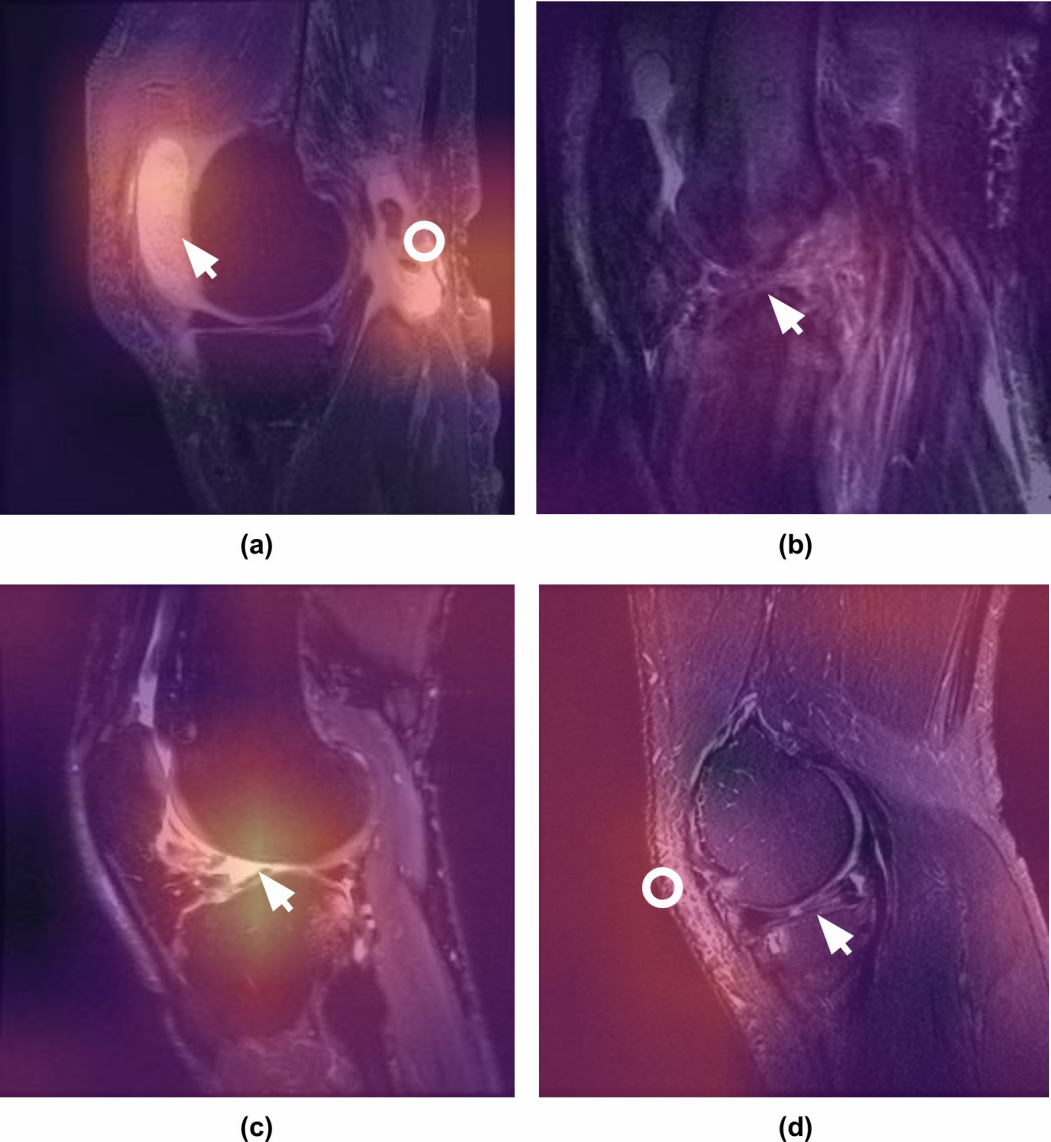
Class activation mappings for MRNet interpretation. Class activation mappings (CAMs) highlight which pixels in the images are important for the model’s classification decision. One of the board-certified musculoskeletal radiologists annotated the images (white arrows and circles) and provided the following captions. (a) Sagittal T2-weighted image of the knee demonstrating large effusion (arrow) and rupture of the gastrocnemius tendon (ring), which were correctly localized by the model and classified as abnormal. Note that the model was not specifically trained to detect these pathologies but was able to recognize the abnormalities based on the contrast with the normal knee examinations. (b) Sagittal T2-weighted image of the knee complicated by a significant motion artifact demonstrating complete anterior cruciate ligament (ACL) tear (arrow), which was correctly classified and localized by the model. Because we hoped to best approximate the clinical practice reality—in which the prevalence of artifacts (i.e. motion, metallic) and other technical noise disrupts interpretation of knee MRI—we did not exclude noisy cases from the training or validation data. (c) Sagittal T2-weighted image of the knee demonstrating complete disruption of the ACL, which was correctly identified by the model as abnormal and classified as ACL tear. The CAM indicates the focus of the model at the abnormal attachment of the ACL (arrow). (d) Sagittal T2-weighted image of the knee demonstrating a complex tear involving the posterior horn of the lateral meniscus (arrow). While the model did classify this examination as abnormal, the CAM indicates that the increased subcutaneous signal (ring) in the anterior/lateral soft tissues contributed to the decision but the meniscal tear did not.

To ensure the MRNet models were learning pertinent features, we generated class activation mappings (CAMs) [30] (Fig 3). To generate a CAM for an image, we computed a weighted average across the 256 CNN feature maps using weights from the classification layer to obtain a 7 × 7 image. The CAM was then mapped to a color scheme, upsampled to 256 × 256 pixels, and overlaid with the original input image. By using parameters from the final layer of the network to weight the feature maps, more predictive feature maps appear brighter. Thus, the brightest areas of the CAMs are the regions that most influence the model’s prediction.

#### Combining MRNet predictions

**Fig 4:**
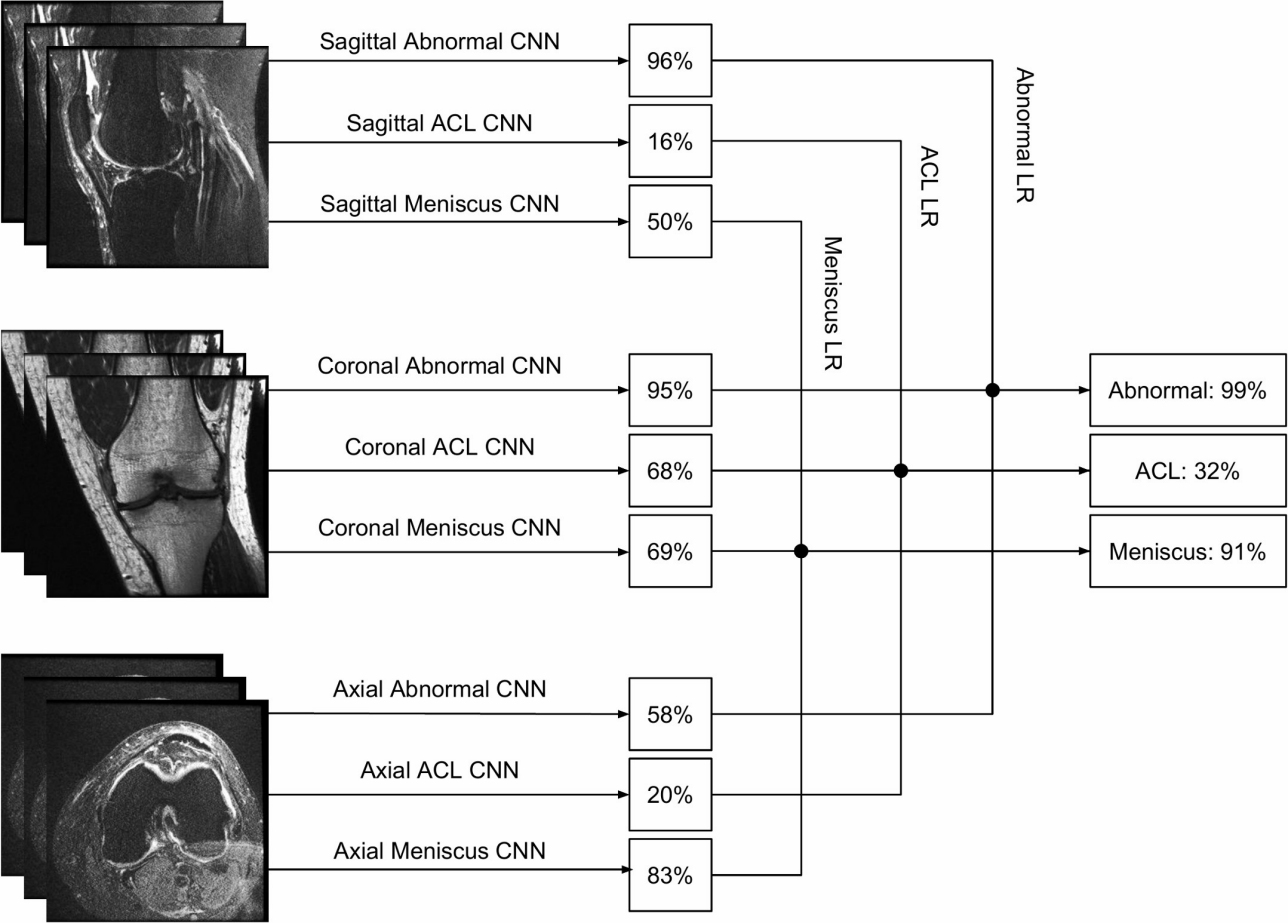
Combining series predictions using logistic regression. Each examination contains 3 types of series: sagittal, coronal, and axial. For each task (abnormality, ACL tear, meniscal tear), we trained a logistic regression classifier to combine the 3 probabilities output by the MRNets to produce a single predicted probability for the exam. The predicted probabilities from an exam in the internal validation set are shown as an example.ACL, anterior cruciate ligament; CNN, convolutional neural network; LR, logistic regression.

Given predictions from the sagittal T2, coronal T1, and axial PD MRNets on the training set, along with their corresponding original labels, we trained a logistic regression to weight the predictions from the 3 series and generate a single output for each exam (Fig 4). The most beneficial series, determined from the coefficients of the fitted logistic regression, were axial PD for abnormalities and meniscal tears and coronal T1 for ACL tears. After training, the logistic regression was applied to the predictions of the 3 MRNets for the internal validation set to obtain the final predictions. We trained 3 logistic regression models in total—1 for each task (detection of abnormalities, ACL tears, and meniscal tears). These models were implemented in Python [24] using the scikit-learn package [31]. For external validation, since there was only 1 series in the dataset, we used the prediction from a single MRNet directly as the final output.

### Evaluation

Reference standard labels were obtained on the internal validation set from the majority vote of 3 practicing board-certified MSK radiologists at a large academic practice (years in practice 6–19 years, average 12 years). The MSK radiologists had access to all DICOM series, the original report and clinical history, and follow-up exams during interpretation. All readers participating in the study used a clinical picture archiving and communication system (PACS) environment (GE Centricity) in a diagnostic reading room, and evaluation was performed on the clinical DICOM images presented on an at least 3-megapixel medical-grade display with a minimum luminance of 1 cd/m^2^, maximum luminance of 400 cd/m^2^, pixel size of 0.2, and native resolution of 1,500 × 2,000 pixels. Exams were sorted in reverse chronological order. Each exam was assigned 3 binary labels for the presence or absence of (1) any abnormality, (2) an ACL tear, and (3) a meniscal tear. Definitions for labels were as follows:

- Abnormality: normal (all images reviewed are free of abnormalities) or abnormal (the abnormal findings in the internal validation set that were not ACL tear or meniscal tear included osteoarthritis, effusion, iliotibial band syndrome, posterior cruciate ligament tear, fracture, contusion, plica, and medial collateral ligament sprain);
- ACL: intact (normal, mucoid degeneration, ganglion cyst, sprain) or tear (low-grade partial tear with <50% of fibers torn, high-grade partial tear with >50% of fibers torn, complete tear) [32];
- Meniscus: intact (normal, degenerative changes without tear, postsurgical changes without tear) or tear (increased signal reaching the articular surface on at least 2 slices or morphologic deformity) [33,34].

Independent of the MSK radiologists, 7 practicing board-certified general radiologists and 2 practicing orthopedic surgeons at Stanford University Medical Center (3–29 years in practice, average 12 years) labeled the internal validation set, blinded to the original reports and labels. These clinical experts’ labels were measured against the reference standard labels established by the consensus of MSK radiologists. The general radiologists were randomly divided into 2 groups, with 4 radiologists in Group 1 and 3 radiologists in Group 2. The 2 orthopedic surgeons were also in Group 1. Group 1 first reviewed the validation set without model assistance, and Group 2 first reviewed the validation set with model assistance. For the reviews with model assistance, model predictions were provided as predicted probabilities of a positive diagnosis (e.g., 0.98 ACL tear). After a washout period of 10 days, Group 1 then reviewed the validation set in a different order with model assistance, and Group 2 reviewed the validation set without model assistance. The Stanford institutional review board approved this study.

### Statistical methods

Performance measures for the model, general radiologists, and orthopedic surgeons included sensitivity, specificity, and accuracy. We also computed the micro-average of these statistics across general radiologists only and across all clinical experts (general radiologists and surgeons). We assessed the model’s performance with the area under the receiver operating characteristic curve (AUC). To assess the variability in estimates, we provide 95% Wilson score confidence intervals [35] for sensitivity, specificity, and accuracy and 95% DeLong confidence intervals for AUC [36,37]. A threshold of 0.5 was used to dichotomize the model’s predictions. The model performance on the external validation set was assessed with the AUC and 95% DeLong confidence intervals.

Because we performed multiple comparisons in this study to assess the model’s performance against that of the practicing general radiologists and also to assess the clinical utility of providing model assistance, we controlled the overall false discovery rate (FDR) at 0.05 [38] and report both unadjusted *p-*values and adjusted *q-*values. Roughly, FDR < 0.05 can be interpreted as the expected proportion (0.05) of false claims of significance across all significant results. Thus, instead of using the unadjusted *p-*value to assess statistical significance, a *q-*value < 0.05 properly accounts for these multiple comparisons. To assess model performance against that of general radiologists, we used a 2-sided Pearson’s chi-squared test to evaluate whether there were significant differences in specificity, sensitivity, and accuracy between the model and the micro-average of general radiologists. The orthopedic surgeons were not included in this comparison.

We assessed the clinical utility of providing model predictions to clinical experts by testing whether the performance metrics across all 7 general radiologists and 2 orthopedic surgeons increased when they were provided model assistance. There is natural variability when a clinical expert evaluates the same knee MRI study at different times, so it is not unexpected that a clinical expert’s performance metrics will be slightly better or slightly worse when tested on two occasions, regardless of model assistance. Thus, we performed robust hypothesis tests to assess if the clinical experts (as a group) demonstrated statistically significant improvement with model assistance. We used a 1-tailed *t* test on the change (difference) in performance metrics for the 9 clinical experts for all 3 labels. To assess whether these findings were dependent specifically on the orthopedic surgeons’ improvement, we performed a sensitivity analysis: we repeated the 1-tailed *t* test on the change in performance metrics across only the general radiologists, excluding the orthopedic surgeons, to determine whether there was still significant improvement.

The exact Fleiss kappa [39,40] is reported to assess the level of agreement of the 3 MSK radiologists, whose majority vote was used for the reference standard labels. Additionally, to assess if model assistance may improve inter-rater reliability, we report the exact Fleiss kappa of the set of 9 clinical experts with and without model assistance for each of the 3 tasks.

All statistical analyses were completed in the R environment for statistical computing [41], using the irr, pROC, binom, and qvalue packages [38,42–44], and R code was provided with submission.

## Results

The inter-rater agreement on the internal validation set among the 3 MSK radiologists, measured by the exact Fleiss kappa score, was 0.508 for detecting abnormalities, 0.800 for detecting ACL tears, and 0.745 for detecting meniscal tears.

### Model performance

**Fig 5:**
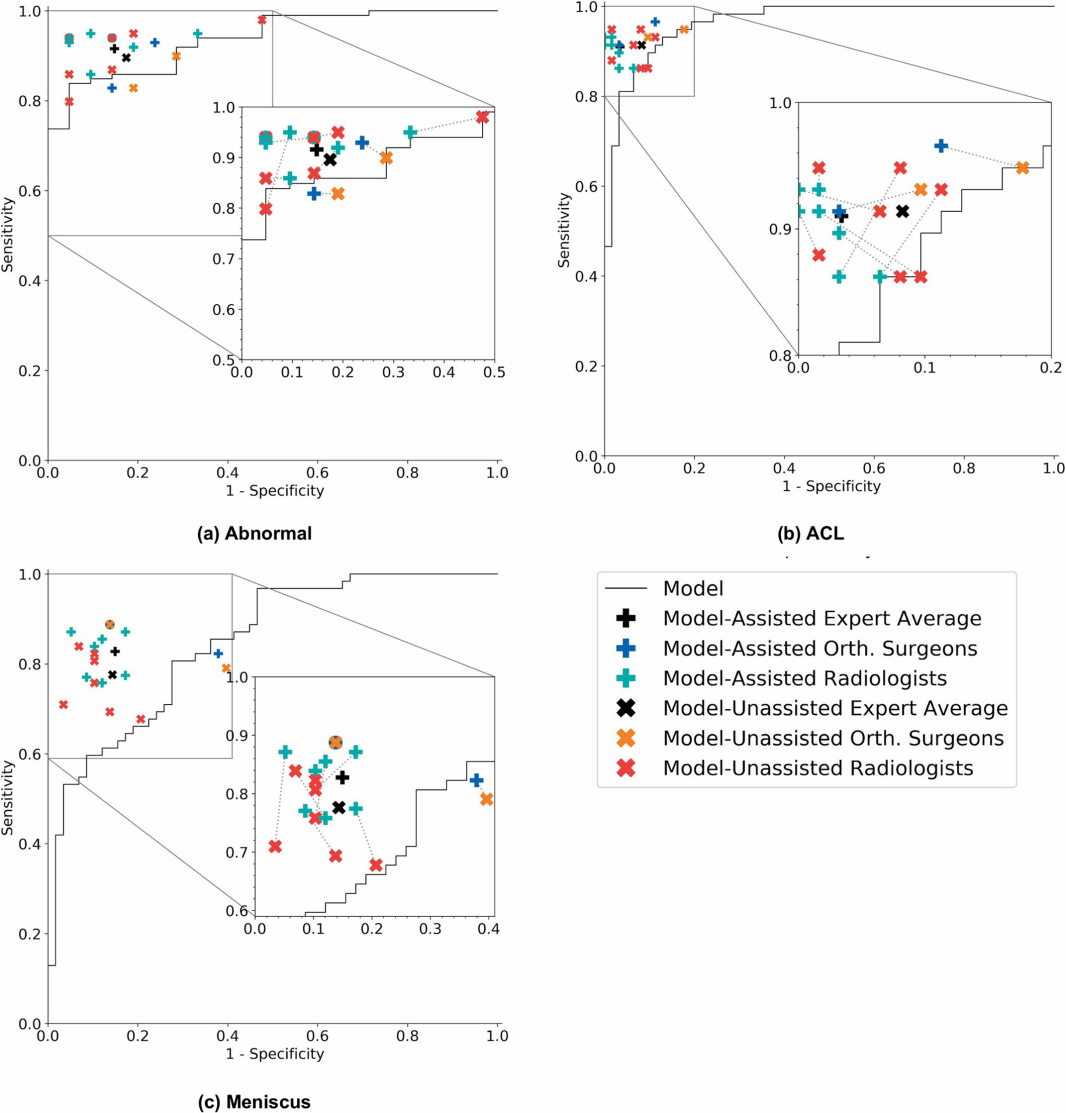
Receiver operating characteristic curves of the model and operating points of unassisted and assisted clinical experts. Each plot illustrates the receiver operating characteristic (ROC) curve of the algorithm (black curve) on the validation set for (a) abnormality, (b) anterior cruciate ligament (ACL) tear, and (c) meniscus tear. The ROC curve is generated by varying the discrimination threshold (used to convert the output probabilities to binary predictions). Individual clinical expert (specificity, sensitivity) points are also plotted, where the red x’s represent model-unassisted general radiologists, the orange x’s represent model-unassisted orthopedic surgeons, the green plus signs represent model-assisted general radiologists, and the blue plus signs represent model-assisted orthopedic surgeons. We also plot the macro-average of the model-unassisted clinical experts (black x’s) and the macro-average of the model-assisted clinical experts (black plus signs). Each unassisted clinical expert operating point is connected to its corresponding model-assisted operating point with a dashed line.

For abnormality detection, ACL tear detection, and meniscal tear detection, the model achieved AUCs of 0.937 (95% CI 0.895, 0.980), 0.965 (95% CI 0.938, 0.993), and 0.847 (95% CI 0.780, 0.914), respectively (Fig 5). In detecting abnormalities, there were no significant differences in the performance metrics of the model and general radiologists (Table 2). The model specificity for abnormality detection was lower than the micro-average of general radiologists, at 0.714 (95% CI 0.500, 0.862) and 0.844 (95% CI 0.776, 0.893), respectively. The model achieved a sensitivity of 0.879 (95% CI 0.800, 0.929) and accuracy of 0.850 (95% CI 0.775, 0.903), while the general radiologists achieved a sensitivity of 0.905 (95% CI 0.881, 0.924) and accuracy of 0.894 (95% CI 0.871, 0.913) (Table 2).

**Table 2:** Comparison of model and general radiologists on the validation set.

Prediction	Specificity (95% CI)	*p-*Value *q-*value	Sensitivity (95% CI)	*p-*Value *q-*value	Accuracy (95% CI)	*p-*Value *q-*value
**Abnormality**						
Model, threshold = 0.5	0.714 (0.500, 0.862)	—	0.879 (0.800, 0.929)	—	0.850 (0.775, 0.903)	—
Unassisted general radiologist micro-average	0.844 (0.776, 0.893)	0.247 0.344	0.905 (0.881, 0.924)	0.528 0.620	0.894 (0.871, 0.913)	0.201 0.301
**ACL tear**						
Model, threshold = 0.5	0.968 (0.890, 0.991)	—	0.759 (0.635, 0.850)	—	0.867 (0.794, 0.916)	—
Unassisted general radiologist micro-average	0.933 (0.906, 0.953)	0.441 0.566	0.906 (0.874, 0.931)	0.002 0.019	0.920 (0.900, 0.937)	0.075 0.173
**Meniscal tear**						
Model, threshold = 0.5	0.741 (0.616, 0.837)	—	0.710 (0.587, 0.808)	—	0.725 (0.639, 0.797)	—
Unassisted general radiologist micro-average	0.882 (0.847, 0.910)	0.003 0.019	0.820 (0.781, 0.853)	0.504 0.619	0.849 (0.823, 0.871)	0.015 0.082

The model was compared to unassisted general radiologists in detection of abnormality, anterior cruciate ligament (ACL) tear, and meniscal tear on a validation set of 120 knee MRI exams on which the majority vote of 3 musculoskeletal radiologists serves as the reference standard. A threshold of 0.5 was used to convert model probabilities to binary predictions before computing specificity, sensitivity, and accuracy. We use 95% Wilson score confidence intervals to estimate the variability in specificity, sensitivity, and accuracy estimates. We conducted a 2-sided Pearson’s chi-squared test to evaluate whether there was a difference between the model and the micro-average of unassisted general radiologists. For each task and metric, we report both unadjusted *p-*values and adjusted *q-*values from this test. A *q-*value < 0.05 indicates statistical significance.

The model was highly specific for ACL tear detection, achieving a specificity of 0.968 (95% CI 0.890, 0.991), which is higher than the micro-average of general radiologists, at 0.933 (95% CI 0.906, 0.953), but this difference was not statistically significant (Table 2). General radiologists achieved significantly higher sensitivity than the model in detecting ACL tears (*p-*value = 0.002, *q-*value = 0.019); the micro-average general radiologist sensitivity was 0.906 (95% CI 0.874, 0.931), while the model achieved a sensitivity of 0.759 (95% CI 0.635, 0.850). The general radiologists also achieved significantly higher specificity in detecting meniscal tears (*p-*value = 0.003, *q-*value = 0.019), with a specificity of 0.892 (95% CI 0.858, 0.918) compared to a specificity of 0.741 (95% CI 0.616, 0.837) for the model. There were no other significant differences in the performance metrics (Table 2). Summary performance metric estimates and confidence intervals can be found in Table 2, and individual performance metrics for the 7 board-certified general radiologists and 2 orthopedic surgeons in this study can be found in S2 Table.

### Clinical utility of model assistance

**Fig 6:**
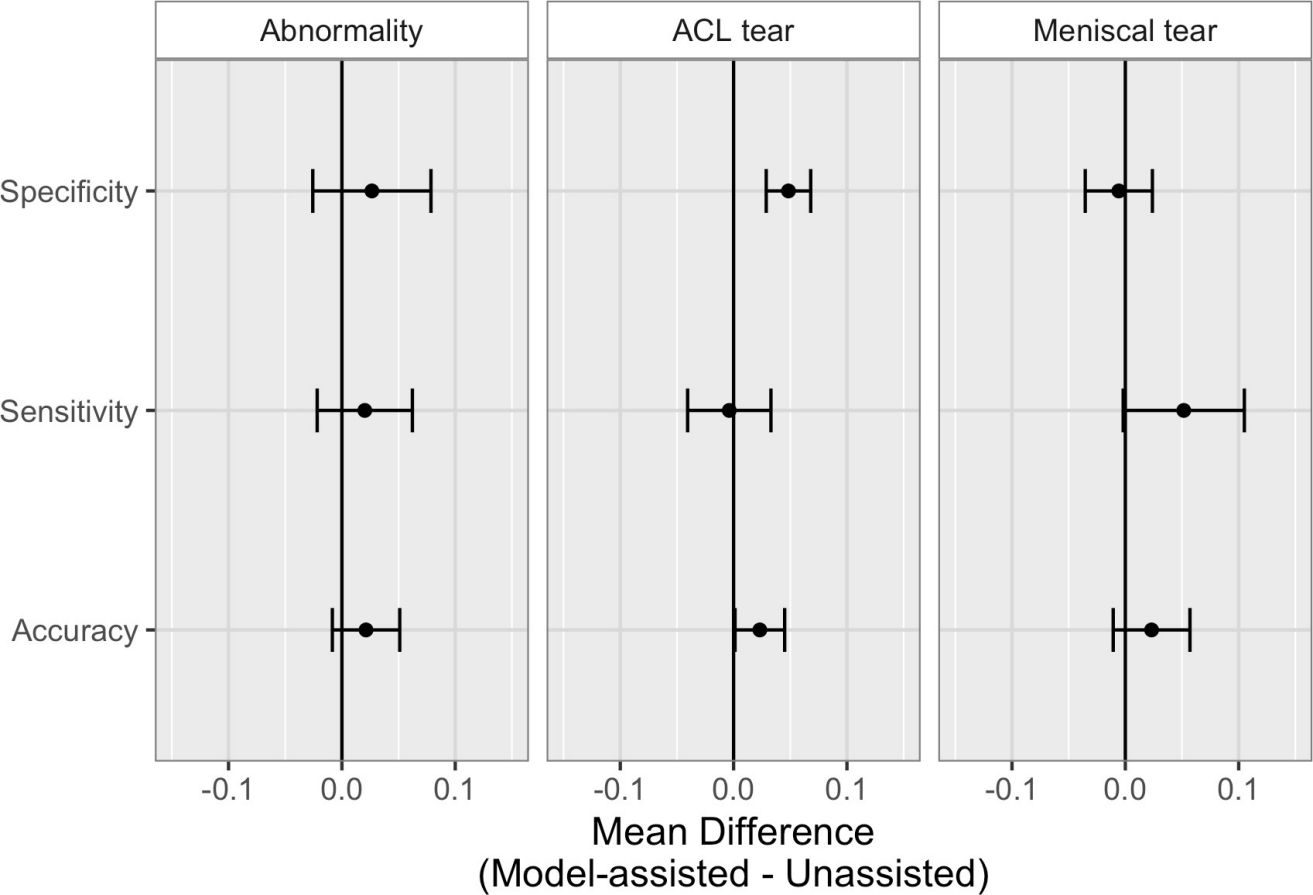
Comparison of unassisted and model-assisted performance metrics of clinical experts on the validation set. Mean differences (with 95% CI error bars) in clinical experts’ performance metrics (model-assisted minus unassisted) for abnormality, anterior cruciate ligament (ACL) tear, and meniscal tear detection. Numerical values are provided in Table 3, and individual values provided in S2 Table.

The clinical utility of providing model predictions to clinical experts during the labeling process is illustrated in Fig 6, and numerical values provided in Table 3. When clinical experts were provided model assistance, there was a statistically significant increase in the clinical experts’ specificity in identifying ACL tears (*p-*value < 0.001, *q-*value = 0.006). The mean increase in ACL specificity was 0.048 (4.8%), and since the validation set contained 62 exams that were negative for ACL tear, this increase in specificity in the optimal clinical setting would mean potentially 3 fewer patients sent to surgery for suspected ACL tear unnecessarily. Though it appeared that model assistance also significantly increased the clinical experts’ accuracy in detecting ACL tears (*p-*value = 0.020) and sensitivity in detecting meniscus tears (*p-*value = 0.028), these findings were no longer significant after adjusting for multiple comparisons by controlling the FDR (*q-*values = 0.092 and 0.110, respectively). There were no other statistically significant improvements to clinical experts’ performance with model assistance. Individual results, unadjusted *p-*values, and adjusted *q-*values are provided in S3 Table.

**Table 3:** Comparison of unassisted and model-assisted performance metrics of clinical experts on the validation set.

Metric	Abnormality		ACL tear		Meniscal tear	
	Mean difference (95% CI)	*p-*Value *q-*value	Mean difference (95% CI)	*p-*Value *q-*value	Mean difference (95% CI)	*p-*Value *q-*value
Specificity	0.026 (−0.026, 0.079)	0.138 0.248	0.048 (0.029, 0.068)	<0.001 0.006	−0.006 (−0.035, 0.024)	0.667 0.692
Sensitivity	0.020 (−0.022, 0.062)	0.150 0.253	−0.004 (−0.041, 0.033)	0.592 0.639	0.052 (−0.002, 0.105)	0.028 0.110
Accuracy	0.021 (−0.008, 0.051)	0.069 0.173	0.023 (0.001, 0.045)	0.020 0.092	0.023 (−0.011, 0.057)	0.077 0.173

Mean differences (95% CIs) in clinical experts’ performance metrics (model-assisted minus unassisted) for abnormality, anterior cruciate ligament (ACL) tear, and meniscal tear detection. Increases in performance when provided model assistance were assessed with a 1-tailed *t* test on the individual differences; both unadjusted *p-*values and adjusted *q-*values are reported. A *q-*value < 0.05 indicates statistical significance. Individual differences in performance metrics provided in S2 Table.

To determine whether the statistically significant improvement in specificity in identifying ACL tears with model assistance was dependent on the orthopedic surgeons’ performance metrics, we assessed the improvement of general radiologists only, excluding orthopedic surgeons. This sensitivity analysis confirmed that even among only general radiologists, there was a significant increase in specificity in identifying ACL tears (*p-*value = 0.003, *q-*value = 0.019; see S4 Table). Additionally, we computed Fleiss kappa for the 9 clinical experts with and without model assistance, and while we did not assess statistical significance, we observed that model assistance increased the Fleiss kappa measure of inter-rater reliability for all 3 tasks. With model assistance, the Fleiss kappa measure for abnormality detection increased from 0.571 to 0.640, for ACL tear detection it increased from 0.754 to 0.840, and for meniscal tear detection it increased from 0.526 to 0.621.

### External validation

The MRNet trained on Stanford sagittal T2-weighted series and Stanford ACL tear labels achieved an AUC of 0.824 (95% CI 0.757, 0.892) on the Štajduhar et al. validation set with no additional training. Additionally, we trained 3 MRNets starting from ImageNet weights on the Štajduhar et al. training set with different random seeds. We selected the MRNet with the lowest average loss on the tuning set and then evaluated this model on the validation set. This model achieved an AUC of 0.911 (95% CI 0.864, 0.958) on the Štajduhar et al. validation set. Štajduhar et al. recorded an AUC of 0.894 for their best model, a semi-automated approach using support vector machines, although it was evaluated using a 10-fold cross-validation scheme [23]. MRNet took less than 30 minutes to train on and less than 2 minutes to evaluate the Štajduhar et al. dataset with an NVIDIA GeForce GTX 12GB GPU.

## Discussion

The purpose of this study was to design and evaluate a deep learning model for classifying pathologies on knee MRI and to compare performance to human clinical experts both with and without model assistance during interpretation in a crossover design. Our results demonstrate that a deep learning approach can achieve high performance in clinical classification tasks on knee MR, with AUCs for abnormality detection, ACL tear detection, and meniscus tear detection of 0.937 (95% CI 0.895, 0.937), 0.965 (95% CI 0.938, 0.965), and 0.847 (95% CI 0.780, 0.847), respectively. Notably, the model achieved high specificity in detecting ACL tears on the internal validation set, which suggests that such a model, if used in the clinical workflow, may have the potential to effectively rule out ACL tears. On an external dataset using T1-weighted instead of T2-weighted series and a different labeling convention for ACL injury, the same ACL tear model achieved an AUC of 0.824 (95% CI 0.757, 0.892). Retraining on the external dataset improved the AUC to 0.911 (95% CI 0.864, 0.958). Our deep learning model achieved state-of-the-art results on the external dataset, but only after retraining. It remains to be seen if the model would better generalize to an external dataset with more MRI series and a more similar MRI protocol. We also found that providing the deep learning model predictions to human clinical experts as a diagnostic aid resulted in significantly higher specificities in identifying ACL tears. Finally, in contrast to the human experts, who required more than 3 hours on average to completely review 120 exams, the deep learning model provided all classifications in under 2 minutes. Our results suggest that deep learning can be successfully applied to advanced MSK MRI to generate rapid automated pathology classifications and that the output of the model may improve clinical interpretations.

There are many exciting potential applications of an automated deep learning model for knee MRI diagnosis in clinical practice. For example, the model described could be immediately applied for diagnostic worklist prioritization, wherein exams detected as abnormal could be moved ahead in the image interpretation workflow, and those identified as normal could be automatically assigned a preliminary reading of “normal.” With its high negative predictive value for abnormalities, the model could lead to quick preliminary feedback for patients whose exams come back as “normal.” Additionally, providing rapid results to the ordering clinician could improve disposition in other areas of the healthcare system. In this work we noticed that specificity for detecting ACL tears improved for both general radiologists and orthopedic surgeons, which implies that this model could help reduce unnecessary additional testing and surgery. Automated abnormality prediction and localization could help general radiologists or even non-radiologist clinicians (orthopedic surgeons) interpret medical imaging for patients at the point of care rather than waiting for specialized radiologist interpretation, which could aid in efficient interpretation, reduce errors, and help standardize quality of diagnoses when MSK specialist radiologists are not readily available. Ultimately, more studies are necessary to evaluate the optimal integration of this model and other deep learning models in the clinical setting. However, our results provide early support for a future where deep learning models may play a significant role in assisting clinicians and healthcare systems.

To examine the effect that a deep learning model may have on the interpretation performance of clinicians, our study deliberately recruited general radiologists to interpret knee MRI exams with and without model predictions. We found a statistically significant improvement in specificity for the ACL tear detection task with model assistance and, though not statistically significant, increased accuracy for ACL tear detection and increased sensitivity for meniscal tear detection. For both general radiologists and non-radiologist clinicians (orthopedic surgeons), we found improved sensitivity and/or specificity across all 3 tasks with model assistance (Fig 5; Table 3), although the group of surgeons was too small for formal analysis. Importantly, model assistance also resulted in higher inter-rater reliability among clinical experts for all 3 tasks, with higher Fleiss kappa measures with model assistance than without. To our knowledge, this is the first study to explore providing outputs of deep learning models to assist radiologists and non-radiologist clinicians in the task of image interpretation. More work will be needed to understand whether and how deep learning models could optimize the interpretation performance of practicing radiologists and non-radiologist clinicians.

A difficulty in deep learning for medical imaging is curating large datasets containing examples of the wide variety of abnormalities that can occur on a given imaging examination to train an accurate classifier, which is a strategy we employed for detecting ACL and meniscal tears. However, our other classification task was to distinguish “normal” from “abnormal” with the intention that if the model could learn the range of normal for a given population of knee MRI exams, then theoretically any abnormality, no matter how rare, could be detected by the model. An example is shown in Fig 3A of a relatively uncommon but serious complete rupture of the gastrocnemius tendon, which was correctly classified and localized as “abnormal” by the model, despite the fact that there were no other examples of this specific abnormality in the abnormal training data. It is possible that with a binary approach and enough “normal” training data, a model could detect any abnormality, no matter how uncommon. However, more work is needed to explore whether subtler abnormalities would require specific training data.

This study has limitations. Our validation set ground truth was not governed strictly by surgical confirmation in all cases. The deep learning model described was developed and trained on MRI data from 1 large academic institution. While MRNet performed well on the external validation set without additional training (AUC 0.824), we saw a substantial improvement (AUC 0.911) after training on the external dataset. This finding suggests that achieving optimal model performance may require additional model development using data more similar to what the model is likely to see in practice. More research is needed to determine if models trained on larger and multi-institutional datasets can achieve high performance without retraining. Power to detect statistically significant gains in clinical experts’ performance with model assistance was limited by the size of the panel, and a larger study that includes more clinical experts as well as more MRI exams may detect smaller gains in utility. Nevertheless, we have shown that even in this small set of clinical experts, providing model predictions significantly increased ACL tear detection specificity, even after correcting for multiple comparisons.

In conclusion, we developed a deep learning model that achieves high performance in clinical classification tasks on knee MRI and demonstrated the benefit, in a retrospective experiment, of providing model predictions to clinicians during the diagnostic imaging task. Future studies are needed to improve the performance and generalizability of deep learning models for MRI and to determine the effect of model assistance in the clinical setting.

## Supporting information

S1 Code MRNet implementation for external validation. (ZIP)

S2 Code Statistical analysis. (RMD)

S1 Table Magnetic resonance imaging settings and parameters for the Stanford musculoskeletal knee protocol. (DOCX)

S2 Table Comparison of individual unassisted and model-assisted clinical experts on the validation set. (DOCX)

S3 Table Comparison of unassisted and model-assisted performance metrics of clinical experts on the validation set. (DOCX)

S4 Table Sensitivity analysis: Comparison of unassisted and model-assisted performance metrics of general radiologists on the validation set. (DOCX)

## Abbreviations

ACL: anterior cruciate ligament
AUC: area under the receiver operating characteristic curve
CAM: class activation mapping
CNN: convolutional neural network
FDR: false discovery rate
MRI: magnetic resonance imaging
MSK: musculoskeletal
PD: proton density
