# Supplementary material for: Deep-learning-assisted diagnosis for knee magnetic resonance imaging: Development and retrospective validation of MRNet
Source: PLoS Med. 2018 Nov 27;15(11):e1002699. doi: 10.1371/journal.pmed.1002699 (PMC6258509; doi:10.1371/journal.pmed.1002699)
Supplement: S1 Table — (DOCX) [file pmed.1002699.s003.docx]

|  | **Repetition time (ms)** | **Echo time (ms)** | **Flip angle (degrees)** | **Pixel bandwidth (Hz)** | **ETL** | **NEX** | **FOV** | **Sect thick (mm)** | **Sect gap (mm)** | **Number of sections** | **Acquisition matrix size** | **Reconstructed matrix size** | **Bit depth** |
| --- | --- | --- | --- | --- | --- | --- | --- | --- | --- | --- | --- | --- | --- |
| **3T magnet** | | | | | | | | | | | | | |
| Cor T1 | 855 | 15 | 142 | 195.312 | 1 | 1.5 | 15 | 2.5 | 0 | 44 | 416 x 224 | 512 x 512 | 16 |
| Cor PD FS | 4788 | 36 | 142 | 195.312 | 9 | 1 | 15 | 2.5 | 0 | 44 | 384 x 224 | 512 x 512 | 16 |
| Sag PD | 3816 | 37 | 142 | 195.312 | 9 | 1 | 15 | 2.5 | 0 | 42 | 384 x 224 | 512 x 512 | 16 |
| Sag T2 FS | 5599 | 54 | 142 | 195.312 | 11 | 2 | 15 | 2.5 | 0 | 42 | 384 x 192 | 512 x 512 | 16 |
| Ax PD FS | 4649 | 35 | 142 | 195.312 | 9 | 1.5 | 15 | 3 | 0.3 | 44 | 512 x 224 | 512 x 512 | 16 |
| **1.5T magnet** | | | | | | | | | | | | | |
| Cor T1 | 716 | 18 | 90 | 81.3672 | 1 | 1 | 16 | 4 | 1 | 20 | 512 x 192 | 512 x 512 | 16 |
| Cor T2 FS | 4516 | 58 | 90 | 81.3672 | 12 | 1 | 16 | 4 | 1 | 20 | 448 x 192 | 512 x 512 | 16 |
| Sag PD | 3066 | 16 | 90 | 81.3672 | 6 | 1 | 16 | 3 | 1 | 24 | 512 x 192 | 512 x 512 | 16 |
| Sag T2 FS | 3266 | 54 | 90 | 81.3672 | 12 | 1 | 16 | 3.5 | 0.5 | 24 | 448 x 192 | 512 x 512 | 16 |
| Ax PD FS | 4150 | 12 | 90 | 81.3672 | 8 | 1 | 13 | 3.5 | 0.5 | 30 | 256 x 192 | 256 x 256 | 16 |

**S1 Table. Magnetic resonance settings and parameters for the Stanford musculoskeletal knee protocol.**
