# Supplementary material for: Deep-learning-assisted diagnosis for knee magnetic resonance imaging: Development and retrospective validation of MRNet
Source: PLoS Med. 2018 Nov 27;15(11):e1002699. doi: 10.1371/journal.pmed.1002699 (PMC6258509; doi:10.1371/journal.pmed.1002699)
Supplement: S2 Table — (DOCX) [file pmed.1002699.s004.docx]

|  | Specificity  (95% CI) | Sensitivity  (95% CI) | Accuracy  (95% CI) |
| --- | --- | --- | --- |
| Unassisted general radiologist 1 | **0.857**  **(0.654, 0.950)** | 0.869  (0.788, 0.922) | 0.867  (0.794, 0.916) |
| Assisted general radiologist 1 | 0.810  (0.600, 0.923) | **0.919**  **(0.849, 0.958)** | **0.900**  **(0.833, 0.942)** |
| Unassisted general radiologist 2 | 0.952  (0.773, 0.992) | 0.939  (0.874, 0.972) | 0.942  (0.884, 0.971) |
| Assisted general radiologist 2 | 0.952  (0.773, 0.992) | 0.939  (0.874, 0.972) | 0.942  (0.884, 0.971) |
| Unassisted general radiologist 3 | **0.952**  **(0.773, 0.992)** | 0.798  (0.708, 0.865) | 0.825  (0.747, 0.883) |
| Assisted general radiologist 3 | 0.905  (0.711, 0.973) | **0.949**  **(0.887, 0.978)** | **0.942**  **(0.884, 0.971)** |
| Unassisted general radiologist 4 | 0.810  (0.600, 0.923) | **0.949**  **(0.887, 0.978)** | 0.925  (0.864, 0.960) |
| Assisted general radiologist 4 | **0.857**  **(0.654, 0.950)** | 0.939  (0.874, 0.972) | 0.925  (0.864, 0.960) |
| Unassisted general radiologist 5 | **0.952**  **(0.773, 0.992)** | 0.859  (0.777, 0.914) | **0.875**  **(0.804, 0.923)** |
| Assisted general radiologist 5 | 0.905  (0.711, 0.973) | 0.859  (0.777, 0.914) | 0.867  (0.794, 0.916) |
| Unassisted general radiologist 6 | 0.524  (0.324, 0.717) | **0.980**  **(0.929, 0.994)** | 0.900  (0.833, 0.942) |
| Assisted general radiologist 6 | **0.667**  **(0.454, 0.828)** | 0.949  (0.887, 0.978) | 0.900  (0.833, 0.942) |
| Unassisted general radiologist 7 | 0.857  (0.654, 0.950) | **0.939**  **(0.874, 0.972)** | 0.925  (0.864, 0.960) |
| Assisted general radiologist 7 | **0.952**  **(0.773, 0.992)** | 0.929  (0.861, 0.965) | **0.933**  **(0.874, 0.966)** |
| Unassisted orthopedic surgeon 1 | 0.810  (0.600, 0.923) | 0.828  (0.742, 0.890) | 0.825  (0.747, 0.883) |
| Assisted orthopedic surgeon 1 | **0.857**  **(0.654, 0.950)** | 0.828  (0.742, 0.890) | **0.833**  **(0.757, 0.889)** |
| Unassisted orthopedic surgeon 2 | 0.714  (0.500, 0.862) | 0.899  (0.824, 0.944) | 0.867  (0.794, 0.916) |
| Assisted orthopedic surgeon 2 | **0.762**  **(0.549, 0.894)** | **0.929**  **(0.861, 0.965)** | **0.900**  **(0.833, 0.942)** |

**S2a Table. Comparison of Individual Unassisted and Assisted Clinical Experts in Abnormality Detection on the Validation Set.**

Radiologists 1-4 completed unassisted first and radiologists 5-7 completed assisted first. Orthopedic surgeons 1 and 2 both completed unassisted first. We used 95% Wilson score confidence intervals to estimate the variability in specificity, sensitivity, and accuracy. Bold text indicates better performance.

|  | Specificity  (95% CI) | Sensitivity  (95% CI) | Accuracy  (95% CI) |
| --- | --- | --- | --- |
| Unassisted general radiologist 1 | 0.903  (0.805, 0.955) | 0.862  (0.751, 0.928) | 0.883  (0.814, 0.929) |
| Assisted general radiologist 1 | **0.984**  **(0.914, 0.997)** | **0.914**  **(0.814, 0.963)** | **0.950**  **(0.895, 0.977)** |
| Unassisted general radiologist 2 | 0.984  (0.914, 0.997) | 0.879  (0.771, 0.940) | 0.933  (0.874, 0.966) |
| Assisted general radiologist 2 | **1.000**  **(0.942, 1.000)** | **0.914**  **(0.814, 0.963)** | **0.958**  **(0.906, 0.982)** |
| Unassisted general radiologist 3 | 0.887  (0.785, 0.944) | **0.931**  **(0.836, 0.973)** | **0.908**  **(0.843, 0.948)** |
| Assisted general radiologist 3 | **0.935**  **(0.846, 0.975)** | 0.862  (0.751, 0.928) | 0.900  (0.833, 0.942) |
| Unassisted general radiologist 4 | 0.935  (0.846, 0.975) | 0.914  (0.814, 0.963) | 0.925  (0.864, 0.960) |
| Assisted general radiologist 4 | **1.000**  **(0.942, 1.000)** | **0.931**  **(0.836, 0.973)** | **0.967**  **(0.917, 0.987)** |
| Unassisted general radiologist 5 | 0.919  (0.825, 0.965) | 0.862  (0.751, 0.928) | 0.892  (0.823, 0.936) |
| Assisted general radiologist 5 | **0.968**  **(0.890, 0.991)** | **0.897**  **(0.792, 0.952)** | **0.933**  **(0.874, 0.966)** |
| Unassisted general radiologist 6 | 0.919  (0.825, 0.965) | **0.948**  **(0.859, 0.982)** | **0.933**  **(0.874, 0.966)** |
| Assisted general radiologist 6 | **0.968**  **(0.890, 0.991)** | 0.862  (0.751, 0.928) | 0.917  (0.853, 0.954) |
| Unassisted general radiologist 7 | 0.984  (0.914, 0.997) | **0.948**  **(0.859, 0.982)** | **0.967**  **(0.917, 0.987)** |
| Assisted general radiologist 7 | 0.984  (0.914, 0.997) | 0.931  (0.836, 0.973) | 0.958  (0.906, 0.982) |
| Unassisted orthopedic surgeon 1 | 0.823  (0.710, 0.898) | 0.948  (0.859, 0.982) | 0.883  (0.814, 0.929) |
| Assisted orthopedic surgeon 1 | **0.887**  **(0.785, 0.944)** | **0.966**  **(0.883, 0.990)** | **0.925**  **(0.864, 0.960)** |
| Unassisted orthopedic surgeon 2 | 0.903  (0.805, 0.955) | **0.931**  **(0.836, 0.973)** | 0.917  (0.853, 0.954) |
| Assisted orthopedic surgeon 2 | **0.968**  **(0.890, 0.991)** | 0.914  (0.814, 0.963) | **0.942**  **(0.884, 0.971)** |

**S2b Table. Comparison of Individual Unassisted and Assisted Clinical Experts in ACL Tear Detection on the Validation Set.**

Radiologists 1-4 completed unassisted first and radiologists 5-7 completed assisted first. Orthopedic surgeons 1 and 2 both completed unassisted first. We used 95% Wilson score confidence intervals to estimate the variability in specificity, sensitivity, and accuracy. Bold text indicates better performance.

|  | Specificity  (95% CI) | Sensitivity  (95% CI) | Accuracy  (95% CI) |
| --- | --- | --- | --- |
| Unassisted general radiologist 1 | 0.793  (0.672, 0.877) | 0.677  (0.554, 0.780) | 0.733  (0.648, 0.804) |
| Assisted general radiologist 1 | **0.828**  **(0.711, 0.904)** | **0.774**  **(0.656, 0.860)** | **0.800**  **(0.720, 0.862)** |
| Unassisted general radiologist 2 | **0.897**  **(0.792, 0.952)** | 0.806  (0.691, 0.886) | 0.850  (0.775, 0.903) |
| Assisted general radiologist 2 | 0.828  (0.711, 0.904) | **0.871**  **(0.766, 0.933)** | 0.850  (0.775, 0.903) |
| Unassisted general radiologist 3 | **0.897**  **(0.792, 0.952)** | 0.758  (0.638, 0.848) | 0.825  (0.747, 0.883) |
| Assisted general radiologist 3 | 0.879  (0.771, 0.940) | **0.855**  **(0.747, 0.922)** | **0.867**  **(0.794, 0.916)** |
| Unassisted general radiologist 4 | **0.966**  **(0.883, 0.990)** | 0.710  (0.587, 0.808) | 0.833  (0.757, 0.889) |
| Assisted general radiologist 4 | 0.948  (0.859, 0.982) | **0.871**  **(0.766, 0.933)** | **0.908**  **(0.843, 0.948)** |
| Unassisted general radiologist 5 | 0.862  (0.751, 0.928) | 0.694  (0.570, 0.794) | 0.775  (0.692, 0.841) |
| Assisted general radiologist 5 | **0.914**  **(0.814, 0.963)** | **0.770**  **(0.651, 0.858)** | **0.833**  **(0.757, 0.889)** |
| Unassisted general radiologist 6 | **0.931**  **(0.836, 0.973)** | **0.839**  **(0.728, 0.910)** | **0.883**  **(0.814, 0.929)** |
| Assisted general radiologist 6 | 0.879  (0.771, 0.940) | 0.758  (0.638, 0.848) | 0.817  (0.738, 0.876) |
| Unassisted general radiologist 7 | **0.897**  **(0.792, 0.952)** | **0.823**  **(0.710, 0.898)** | **0.858**  **(0.785, 0.910)** |
| Assisted general radiologist 7 | 0.882  (0.847, 0.910) | 0.820  (0.781, 0.853) | 0.849  (0.823, 0.871) |
| Unassisted orthopedic surgeon 1 | 0.603  (0.475, 0.719) | 0.790  (0.674, 0.873) | 0.700  (0.613, 0.775) |
| Assisted orthopedic surgeon 1 | **0.621**  **(0.492, 0.734)** | **0.823**  **(0.710, 0.898)** | **0.725**  **(0.639, 0.797)** |
| Unassisted orthopedic surgeon 2 | 0.862  (0.751, 0.928) | 0.887  (0.785, 0.944) | 0.875  (0.804, 0.923) |
| Assisted orthopedic surgeon 2 | 0.862  (0.751, 0.928) | 0.887  (0.785, 0.944) | 0.875  (0.804, 0.923) |

**S2c Table. Comparison of Individual Unassisted and Assisted Clinical Experts in Meniscal Tear Detection on the Validation Set.**

Radiologists 1-4 completed unassisted first and radiologists 5-7 completed assisted first. Orthopedic surgeons 1 and 2 both completed unassisted first. We used 95% Wilson score confidence intervals to estimate the variability in specificity, sensitivity, and accuracy. Bold text indicates better performance.
