# Supplementary material for: Deep-learning-assisted diagnosis for knee magnetic resonance imaging: Development and retrospective validation of MRNet
Source: PLoS Med. 2018 Nov 27;15(11):e1002699. doi: 10.1371/journal.pmed.1002699 (PMC6258509; doi:10.1371/journal.pmed.1002699)
Supplement: S3 Table — (DOCX) [file pmed.1002699.s005.docx]

|  | Abnormal | | | ACL Tear | | | Meniscal Tear | | |
| --- | --- | --- | --- | --- | --- | --- | --- | --- | --- |
|  | Spec. | Sens. | Acc. | Spec. | Sens. | Acc. | Spec. | Sens. | Acc. |
| General radiologist 1 | −0.048 | 0.051 | 0.033 | 0.081 | 0.052 | 0.067 | 0.034 | 0.097 | 0.067 |
| General radiologist 2 | 0.000 | 0.000 | 0.000 | 0.016 | 0.034 | 0.025 | −0.069 | 0.065 | 0.000 |
| General radiologist 3 | −0.048 | 0.152 | 0.117 | 0.048 | −0.069 | −0.008 | −0.017 | 0.097 | 0.042 |
| General radiologist 4 | 0.048 | −0.010 | 0.000 | 0.065 | 0.017 | 0.042 | −0.017 | 0.161 | 0.075 |
| General radiologist 5 | −0.048 | 0.000 | −0.008 | 0.048 | 0.034 | 0.042 | 0.052 | 0.077 | 0.058 |
| General radiologist 6 | 0.143 | −0.030 | 0.000 | 0.048 | −0.086 | −0.017 | −0.052 | −0.081 | −0.067 |
| General radiologist 7 | 0.095 | −0.001 | 0.008 | 0.000 | −0.017 | -0.008 | 0.000 | 0.016 | 0.008 |
| Orthopedic surgeon 1 | 0.048 | 0.000 | 0.008 | 0.065 | 0.017 | 0.042 | 0.017 | 0.032 | 0.025 |
| Orthopedic surgeon 2 | 0.048 | 0.030 | 0.033 | 0.065 | −0.017 | 0.025 | 0.000 | 0.000 | 0.000 |
| *p*-value | 0.138 | 0.150 | 0.069 | < 0.001 | 0.592 | 0.020 | 0.667 | 0.028 | 0.077 |
| *q*-value | 0.248 | 0.253 | 0.173 | 0.006 | 0.639 | 0.092 | 0.692 | 0.110 | 0.173 |

**S3 Table. Comparison of Unassisted and Model-Assisted Performance Metrics of Clinical Experts on the Validation Set.**

Differences in clinical experts' performance metrics (model-assisted minus unassisted) for abnormality, ACL tear, and meniscal tear detection. Statistically significant increases in performance when provided model assistance were assessed with a one-tailed *t*-test on these differences; both unadjusted *p*-values and adjusted *q*-values are reported. Abbreviations: Sens. = Sensitivity, Spec. = Specificity, Acc. = Accuracy. A *q*-value < 0.05 indicates statistical significance.
