# Supplementary material for: Deep-learning-assisted diagnosis for knee magnetic resonance imaging: Development and retrospective validation of MRNet
Source: PLoS Med. 2018 Nov 27;15(11):e1002699. doi: 10.1371/journal.pmed.1002699 (PMC6258509; doi:10.1371/journal.pmed.1002699)
Supplement: S4 Table — (DOCX) [file pmed.1002699.s006.docx]

|  | Abnormal | | | ACL Tear | | | Meniscal Tear | | |
| --- | --- | --- | --- | --- | --- | --- | --- | --- | --- |
|  | Spec. | Sens. | Acc. | Spec. | Sens. | Acc. | Spec. | Sens. | Acc. |
| *p*-value | 0.255 | 0.197 | 0.123 | 0.003 | 0.591 | 0.072 | 0.716 | 0.038 | 0.107 |
| *q*-value | 0.344 | 0.301 | 0.237 | 0.019 | 0.639 | 0.173 | 0.716 | 0.129 | 0.222 |

**S4 Table. Sensitivity Analysis: Comparison of Unassisted and Model-Assisted Performance Metrics of General Radiologists on the Validation Set.**

Statistically significant increases in general radiologists' performance when provided model assistance were assessed with a one-tailed *t*-test on the differences (model-assisted minus unassisted) for abnormality, ACL tear, and meniscal tear. Both unadjusted *p*-values and adjusted *q*-values are reported. Differences in general radiologists' performance metrics for abnormality, ACL tear, and meniscal tear detection provided in Table 3. Abbreviations: Sens. = Sensitivity, Spec. = Specificity, Acc. = Accuracy. A q-value < 0.05 indicates statistical significance.
